# Collective Polarization of Cancer Cells at the Monolayer Boundary

**DOI:** 10.3390/mi12020112

**Published:** 2021-01-22

**Authors:** Liu-Yuan Guan, Jian-Qing Lv, De-Qing Zhang, Bo Li

**Affiliations:** Institute of Biomechanics and Medical Engineering, Applied Mechanics Laboratory, Department of Engineering Mechanics, Tsinghua University, Beijing 100084, China; gly16@mails.tsinghua.edu.cn (L.-Y.G.); lvjq18@mails.tsinghua.edu.cn (J.-Q.L.); zdq17@mails.tsinghua.edu.cn (D.-Q.Z.)

**Keywords:** cancer cells, collective polarization, Golgi apparatus, membrane protrusion

## Abstract

Cell polarization, a process depending on both intracellular and intercellular interactions, is crucial for collective cell migration that commonly emerges in embryonic development, tissue morphogenesis, wound healing and cancer metastasis. Although invasive cancer cells display weak cell–cell interactions, they can invade host tissues through a collective mode. Yet, how cancer cells without stable cell–cell junctions polarize collectively to migrate and invade is not fully understood. Here, using a wound-healing assay, we elucidate the polarization of carcinoma cells at the population level. We show that with loose intercellular connections, the highly polarized leader cells can induce the polarization of following cancer cells and subsequent transmission of polarity information by membrane protrusions, leading to gradient polarization at the monolayer boundary. Unlike the polarization of epithelial monolayer where Rac1/Cdc42 pathway functions primarily, our data show that collective polarization of carcinoma cells is predominantly controlled by Golgi apparatus, a disruption of which results in the destruction of collective polarization over a large scale. We reveal that the Golgi apparatus can sustain membrane protrusion formation, polarized secretion, intracellular trafficking, and F-actin polarization, which contribute to collective cancer cell polarization and its transmission between cells. These findings could advance our understanding of collective cancer invasion in tumors.

## 1. Introduction

Collective cell migration occurs in various fundamental biological processes, including embryonic development, tissue renewal, angiogenesis, and tumor spreading [1,2,3,4,5,6,7,8,9]. During collective migration, groups of cells form spatiotemporal patterns and usually migrate more efficiently than isolated cell migration [10,11,12,13,14]. Generally, collective cell migration displays two hallmarks. First, cell–cell connections are mediated by intercellular junction proteins [15], which either directly or indirectly link to the cytoskeleton, providing mechanically robust and dynamic coupling between cells during their movement [16]. Second, in multicellular clusters, the front-to-rear multicellular-scale polarity of cell monolayer gives rise to traction and protrusion force for migration [2,17,18,19].

In multicellular organisms, stable intercellular junctions contribute to maintaining the polarity and integrity of healthy tissues. These cell–cell junctions form compact connections between neighboring cells, and those connections, together with different components of the cytoskeleton system, generate an integrated continuum across the tissue. For example, classical cadherins, such as E-cadherin, form a complex involving β-catenin, α-catenin, and p120-catenin, which binds to F-actin in a mechanical force-dependent mode [20]. With the help of actin-binding protein afadin (AF-6), cell junction molecules could form direct links to the cytoskeleton to regulate cell polarization in early embryonic development [21,22]. Polarized cytoskeleton and activated Cdc42 are essential for the polarity signal transduction of intracellular organelles [23,24,25]. Cell–cell junctions also mediate intercellular communication by allowing the passage of signals between neighboring cells. In normal epithelium, the establishment of cell–cell junctions is implicated in the transmission of cell polarity information. In the wing epithelium of *Drosophila*, for instance, it was reported that the transmission of polarity information could be parallel to a relay from cell to cell [26].

Underlying the functional polarization of normal tissues is a front-to-rear polarization of signaling cascades, among which the activation of Rho GTPases in the Rho family is the predominant pathway throughout the whole process [27,28,29]. At the front of cells, Rac1 and Cdc42 induce cytoskeletal rearrangement, including rapid actin polymerization, which leads to the formation of protrusions such as filopodia and lamellipodia [30,31] and promotes the formation of integrins binding to the extracellular matrix (ECM). At the rear of cells, a distinct signaling pathway involving Rho promotes actomyosin contraction. Other approaches may include but are not limited to intracellular organelles, which locate asymmetrically at positions along the polarity axis of the cell. As a major intracellular organelle, the Golgi apparatus has the capacity to sustain polarized secretion and intracellular trafficking [32,33]. The Golgi biosynthetic pathway is primordial to the secretion of small secretory cargoes, ECM components, and membrane-bound proteins [34]. Furthermore, the Golgi apparatus is intracellularly positioned close to the microtubule organizing center (MTOC). Directly binding to Golgi apparatus, molecular motors interact with microtubules and regulate the movement of small secretory vesicles along the microtubule tracks. Downregulation of cell–cell junctions can lead to the failure of cell polarization and disrupt the transmission of cell polarity, resulting in uncorrelated cell motion. Strikingly distinct from healthy cells, cancer cells naturally exhibit weak cell–cell junctions. Both *in vitro* and *in vivo* experiments have shown that this weak intercellular adhesion favors the invasion of carcinoma cells. Although many invasive tumors, such as breast and lung cancer, are found to display predominantly population-level polarization and collective invasion [35], it remains controversial how cancer cells without stable intercellular junctions achieve collective polarization.

In this study, by combining wound healing assay, Golgi apparatus staining, and F-actin filaments polarization analysis, we identify the mechanism underpinning the collective polarization of cancer cells with low cell–cell junctions. Human breast carcinoma MDA-MB-231 cell monolayers are used as our model system. We demonstrate that MDA-MB-231 cells at the leading edge of the monolayer exhibit a stable, higher degree of polarization than cells located at the monolayer center. Without stable intercellular junctions, polarized leader MDA-MB-231 cells are still able to transmit polarity information and drive the polarization of follower cells through membrane protrusions. We find that the Golgi apparatus pathways predominate over the Rac1/Cdc42 signaling pathways in controlling the cancer cell polarization, in contrast to the polarization of healthy tissues and cells that are primarily controlled by the Rac1/Cdc42 signaling pathways. The polarized Golgi contributes to sustaining polarized secretion, intracellular trafficking, F-actin polarization, and membrane protrusion formation, which are synergistically required in cell polarization at the population level. Our results reveal an unappreciated polarization strategy that cancer cells adopt to migrate and invade collectively.

## 2. Materials and Methods

### 2.1. Cell Generation and Sorting

Human breast carcinoma MDA-MB-231 cell line was bought from China Infrastructure of Cell Line Resource (Beijing, China). MDA-MB-231 cells were transduced with lentivirus carrying the EGFP sequence. Non-tumorigenic mammary epithelial cells MCF10A cells were transduced with lentivirus carrying the mCherry sequence. Positive cells were selected using a FACSARIA III fluorescence-activated cell sorter (BD BioSciences, San Jose, CA, USA). Positive MDA-MB-231 cells were infected with lentivirus carrying *hTUBB*-mCherry sequence. Next, infected MDA-MB-231 cells were selected by mCherry using the cell sorter. The lentiviruses were obtained from Cyagen Biosciences (Santa Clara, CA, USA).

### 2.2. Cell Culture

MDA-MB-231 cells were grown in L15 culture medium supplemented with 10% fetal bovine serum (FBS), 100 U/mL penicillin, and 100 µg/mL streptomycin. MCF-10A cells were grown in DMEM-F12 medium supplemented with 5% horse serum, 20 ng/mL EGF, 0.5 mg/mL hydrocortisone, 100 ng/mL cholera toxin and 10 µg/mL insulin, 100 U/mL penicillin, and 100 µg/mL streptomycin. Cells were maintained at 37 ℃ in a humidified atmosphere with 5% CO_2_.

### 2.3. In Vitro Wound Healing Assay

For the wound healing assay, MDA-MB-231 cells (4 × 10^5^ cells) were plated on 35 mm glass-based confocal dishes. One day after seeding, confluent MDA-MB-231 cells were scratched with a 20 µL pipette tip to establish a wound. After the scratching, the media and dislodged cells were aspirated and replaced by fresh L15 culture medium. Immediately after replacing the media, the cells were imaged with an inverted confocal microscope.

### 2.4. Chemicals and Reagents

Brefeldin A (BFA) (S7046, Selleck, Houston, TX, USA) and ML141 (S7686, Selleck, Houston, TX, USA) dissolved in dimethyl sulfoxide (DMSO) were used 5 µM and 10 µM, respectively. BFA and ML141 treatment was done for 2 h in serum-free media and washed out before the wound healing assay.

### 2.5. Golgi Apparatus Staining

The glass bottom of the confocal dish was coated with fibronectin (FN). A total of 2 × 10^5^ cells were seeded and allowed to attach for 12 h before staining. The reagent CellLight Golgi-RFP (C10593, Thermofisher, Waltham, MA, USA) was added directly to the MDA-MB-231 cells. After incubated overnight, the cells were ready to image the next morning. Hoechst 33342 (H3570, Thermofisher, Waltham, MA, USA), diluted at 1:5000 in PBS, was added and incubated for at least 10 min. Cells were then washed twice, for 3 min each, in prewarmed PBS.

### 2.6. Immunofluorescence Staining

Immunofluorescence microscopy experiments were carried out by fixing the MDA-MB-231 cells with 4% paraformaldehyde in PBS, permeabilizing with 0.1% Triton X-100 in PBS, and blocking with 5% BSA in PBS. Primary antibodies rabbit anti-GM130 (12480S, Cell Signaling Technology, Massachusetts, MA, USA), rabbit anti-E-cadherin (ab40772, Abcam, Cambridge, UK), diluted at 1:200, in 5% BSA in PBS, were incubated overnight and were detected using secondary antibodies donkey anti-rabbit Alexa Fluor 647 (A-21245, Thermofisher, Waltham, MA, USA). Rhodamine phalloidin (R415, Thermofisher, Waltham, MA, USA), diluted at 1:40 in PBS, was incubated for 1 h with the secondary antibodies. DAPI (D1306, Thermofisher, Waltham, MA, USA), diluted at 1:2000 in PBS, was incubated for 10 min.

### 2.7. Time-Lapse Imaging and Image Analysis

Multidimensional acquisitions were performed on an automated inverted confocal microscope (Nikon A1, Tokyo, Japan) equipped with thermal, CO_2_ and humidity control (Okolab). The interval between image acquisitions was 5 min and a typical experiment lasted for 10–15 h. Images were acquired with a 20 × objective. Multi-image stitching was used. To analyze cell polarization, MDA-MB-231 cells were detected by DAPI and Golgi apparatus using the open image analysis software Fiji, v1.53c (http://fiji.sc/Fiji, NIH, USA) and Nikon NIS-Elements AR Software (Nikon).

### 2.8. Time-Lapse Cell Motility Assay and Cell Trajectory Analysis

Incubated with Hoechst 33342, MDA-MB-231 cell nuclei were labeled with fluorescent tag. The interval between image acquisitions was 5 min and a typical experiment lasted for 10–15 h. Cell motility parameters were calculated based on the fluorescent images of cell nuclei using NIS-Element AR Software, v 5.21.01 (Nikon, Tokyo, Japan). Movements of individual cells were traced by tracking the translocation of the cell nuclei using automated tracking options on NIS-Element AR Software [36]. About 50 cells were selected for analyzing time-lapse images.

### 2.9. Alignment Characterization of F-Actin Filaments

The structure tensor method was adopted to quantify the polarization of F-actin filaments [37,38]. After the fluorescent images of F-actin filaments were acquired, they were denoised via a low-pass Gaussian filter of kernel size typically 16 pixels. Then the coarse-grained structure tensors for each pixel were quantified as

(1) $$J_{w}=\left(\begin{array}{cc} {\langle I_{x},I_{x}\rangle }_{w} & {\langle I_{x},I_{y}\rangle }_{w}\\ {\langle I_{y},I_{x}\rangle }_{w} & {\langle I_{y},I_{y}\rangle }_{w} \end{array}\right)$$

where $I_{x}$ and $I_{y}$ denote the partial derivatives of the filtered grayscale images along x and y direction, respectively;

(2) $${\langle f,g\rangle }_{w}=\int w\left(x^{\prime }-x,y^{\prime }-y\right)f\left(x^{\prime },y^{\prime }\right)g\left(x^{\prime },y^{\prime }\right)dx^{\prime }dy^{\prime }$$

with *f* and *g* being arbitrary functions and $w\left(x^{\prime }-x,y^{\prime }-y\right)$, which was treated as the Gaussian filter, representing the weighting function centered at $\left(x,y\right)$. Once the structure tensors were obtained, we calculated the eigenvalues ($\lambda_{\max}^{J}>\lambda_{\min}^{J}$) and the corresponding eigenvectors of the structure tensor $J_{w}$. The local orientation $\theta_{\mathrm{orient}}$ of F-actin filaments could be identified as the orientation of the eigenvector associated with the smallest eigenvalue $\lambda_{\min}^{J}$. $\theta_{\mathrm{orient}}$ within the range (−60°, 60°) facing the wound edge was regarded as polarized F-actin filaments. The polarization degree of F-actin filaments can be evaluated by the relative discrepancy between the two eigenvalues $\lambda_{\max}^{J}$ and $\lambda_{\min}^{J}$ as

(3) $$\lambda_{\mathrm{orient}}=\frac{\lambda_{\max}^{J}-\lambda_{\min}^{J}}{\lambda_{\max}^{J}+\lambda_{\min}^{J}}$$

## 3. Results

### 3.1. Leader Cells Exhibit a Stronger Polarization than Follower Cells

**Figure 1:**
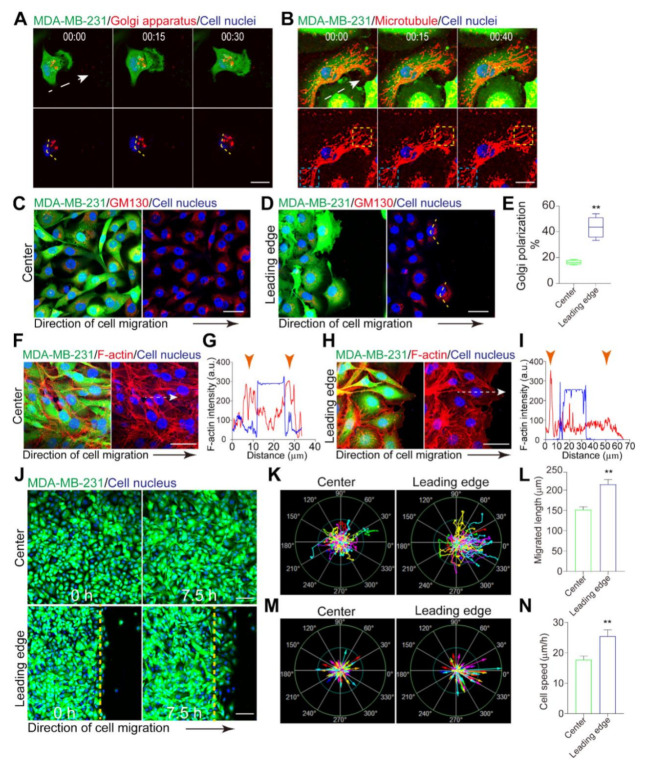
Leader cells exhibit a higher degree of polarization than follower cells. (**A**) Time-lapse images showing a single MDA-MB-231 cell polarization during migration with elapsed time depicted (h:min). Golgi apparatus (red) and cell nuclei (blue) were distributed in the MDA-MB-231 cell (green). Dotted arrow points toward the orientation of cell migration. Scale bar, 20 µm. (**B**) Time-lapse images showing the rearrangement of microtubules in MDA-MB-231 cell during cell migration with elapsed time depicted (h:min). Microtubules (red) and cell nuclei (blue) distribution in MDA-MB-231 cell (green). The yellow dotted boxes indicated the reorganization of microtubules at the front of cell. The blue dotted boxes indicated the disruption of microtubules at the rear of cell. Dotted arrow points toward the orientation of cell migration. Scale bar, 20 µm. (**C**,**D**) MDA-MB-231 cells (green) were stained for the Golgi marker GM130 (red). MDA-MB-231 cells localized at the center (**C**) and at the leading edge (**D**) of the monolayer. Scale bar, 40 µm. (**E**) The ratio of numbers of cells polarized at the center and the leading edge, respectively. (**F**–**I**) Polarization of F-actin. Fluorescence images of F-actin (phalloidin) in migrating MDA-MB-231 cells at the center of the monolayer (**F**) and at the leading edge (**H**). Scale bar, 20 µm. Intensity profile of F-actin along the white dotted arrow in MDA-MB-231 cells at the center (**G**) and at the leading edge (**I**). Orange arrowheads in (**G**,**I**) indicated the boundaries of the selected cells. (**J**) Collective migration of MDA-MB-231 cells. The yellow dotted lines indicated the position of the edge at 0 h. Scale bar, 100 µm. (**K**,**L**) Trajectories (**K**) and trajectory length (**L**) of migrating MDA-MB-231 cells at the center and at the leading edge at 10 h. In (**K**), the green rings denote 225 µm and 450 µm, respectively. In (**K**), the trajectory length was measured at 10 h. (**M**,**N**) Migration velocity vectors and speed of MDA-MB-231 cells at the center and at the leading edge. In (**M**), the green rings denote 36 µm/h and 72 µm/h, respectively. Data were the mean ± SEM. **, *p* < 0.001. *P* values were calculated using each-pair Student’s *t*-test.

MDA-MB-231 cell line is considered a dangerous subtype among multiple types of human breast cancer [39,40]. Cell polarization is an initial step of epithelial cancer metastasis and invasion. To analyze cell polarization, MDA-MB-231 cells and their internal organelles were labeled with different fluorescent tags. Golgi polarization was determined by well-established criteria, where the Golgi apparatus was recognized as a polarized one when it was located within the orientation angle range (−60°, 60°) relative to the cell migration direction (Figure 1A). Microtubule networks were primarily polarized, and their orientation and density were conducive to cell polarity. We observed that MDA-MB-231 cells displayed polarized microtubules protruding toward the orientation of migrating (Figure 1B). Moreover, in the lamellipodia at the front of cells, we found microtubules self-assembled from tubulin dimers. However, the microtubules were buckling and breaking at the cell rear as the cell moved forward (Figure 1B).

Collective cell dynamics give rise to complex alterations in multicellular tissue structures, suggesting collective cell polarization is remarkably different from the polarization of a single cell. To accurately evaluate MDA-MB-231 cell polarization during collective migration, we stained Golgi apparatuses for GM130 to visualize their localization relative to the nucleus. A wound was introduced to MDA-MB-231 cell monolayer to examine how the Golgi apparatus oriented during cell migration. As cells migrated toward the free space, we observed that the Golgi orientation of cells at the monolayer’s leading edge was significantly different from cells located at the center (distance >500 µm from the leader cells) (43.88% vs. 16.44%, *p* < 0.0001) (Figure 1C–E). Moreover, compared with cells located at the center, we found that the subcellular localization of F-actin in migrating cells was altered (Figure 1F–I). It showed that numerous F-actin filaments localized at the rear of cells at the leading boundary, which indicated that they constituted an intracellular asymmetrically of the cytoskeleton. However, there was no observable difference in the intensity of F-actin between the front and rear of cells at the center.

Cell polarization can mediate cellular dynamics and behaviors. We tracked the position of cell nuclei in time-lapse image series for a detailed analysis of cellular behaviors. The results showed that cells at the leading edge statistically underwent a few orientation changes, suggesting that the leader cells performed persistent cell migration (Figure 1J–L). By contrast, cells located at the center displayed random motion. In addition, cells at the leading edge had a higher velocity than the cells at the center (25.42 µm/h vs. 17.72 µm/h, *p* = 0.0024) (Figure 1M,N). Together, these results indicate that the cell polarization at the monolayer boundary is profoundly different from that of isolated cells. Furthermore, the leader cells exhibit a higher degree of polarization than cells located at the center, resulting in a highly heterogeneous polarity across the cell monolayer.

### 3.2. Polarity Transmits by Principal Membrane Protrusions

Due to the intracellular and intercellular cross-talking, the mechanism of collective cell polarization is quite sophisticated. How collective cell polarization and coordination are achieved in cancer cells with low cell–cell junctions is not completely understood. We next decipher the mechanisms underlying the collective polarization of carcinoma cells.

**Figure 2:**
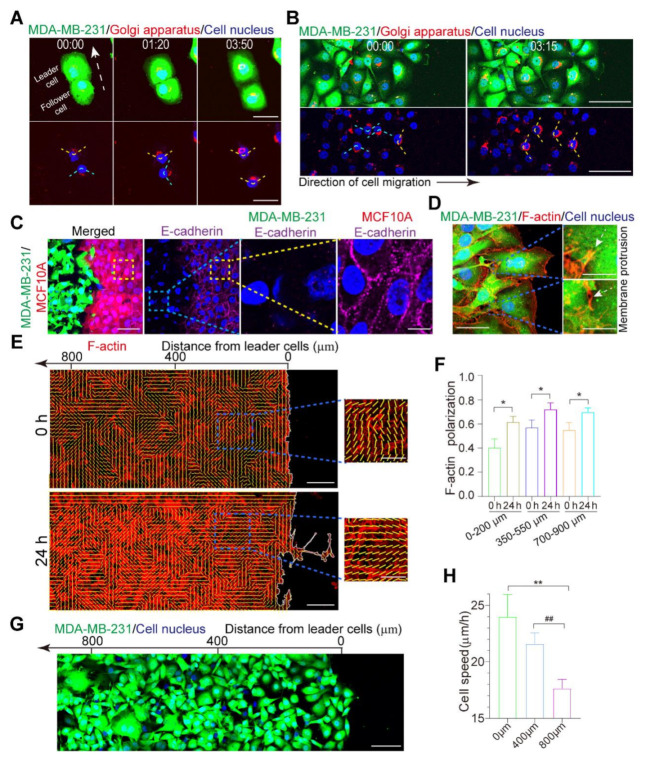
Polarized leader MDA-MB-231 cells drive follower cell polarization. (**A**) Time-lapse images showing the polarization of two cells during migration. Golgi apparatus (red) and cell nuclei (blue) distributed in the MDA-MB-231 cell (green). Dotted arrow pointed toward the orientation of cell migration. Time, h:min. Scale bar, 50 µm. (**B**) Time-lapse images showing collective MDA-MB-231 cell polarization during migration with elapsed time depicted (h:min). Golgi apparatus (red) and cell nuclei (blue) distributed in the MDA-MB-231 cell (green). Scale bar, 100 µm. (**C**) Immunofluorescence localization of E-cadherin in MDA-MB-231 and MCF-10A cell monolayer. MDA-MB-231 (green), MCF10A (red), E-cadherin (purple), and nuclei stained with DAPI (blue) were distinguished with different fluorescences. Scale bar, 50 µm. Boxes indicated magnified areas shown in the right image. Scale bar, 10 µm. (**D**) Membrane protrusions between the leader cells and follower cells. Scale bar, 20 µm. Blue dotted boxes showed local membrane protrusions. Scale bar, 5 µm. (**E**) F-actin filaments (red) were reorganized in wound healing assay at 0 h and 24 h. Scale bar, 100 µm. Blue dotted boxes showed a group of F-actin polarized in MDA-MB-231 cells. Scale bar, 50 µm. (**F**) The ratio of polarized F-actin in MDA-MB-231 cells located at the regions with different distances from the leader cells, including (0–200 µm), (350–550 µm) and (700–900 µm). (**G**) MDA-MB-231 cell migration oriented to the wound. Scale bar, 100 µm. (**H**) Cell speed of migrating MDA-MB-231 cells at a different distance from the edge, 0 µm, 400 µm, and 800 µm. Data were the mean ± SEM. *, ** and ##, *p* < 0.005, *p* < 0.001 and *p* < 0.001. *P* values were calculated using each-pair Student’s *t*-test.

To identify the elements regulating collective cell polarization, time-series data of two MDA-MB-231 cells during migration and wound healing processes were collected (Figure 2A,B). We observed that the polarization of the follower cells relied mainly on the polarization of leader cells. The orientation of the follower cell gradually became aligned with the leader cells. To evaluate the cell–cell junctions, we examined E-cadherin in MDA-MB-231 and non-tumorigenic human breast epithelial cells (MCF10A) by immunostaining (Figure 2C). Remarkably, immunostaining results showed that the cell–cell junctions of MDA-MB-231 cells were lower than that of MCF10A cells. However, at the front edge of the monolayer, supracellular fingers emerged, which was caused by a highly protrusive cell that acted as a leader cell. Moreover, we found that the follower cells generated membrane protrusion structures linked to the leader cells (Figure 2D). The formation and extension of local membrane protrusions resulted from the polymerizations of actin and membrane ruffles that arose on the dorsal surfaces. The principal membrane protrusions could drive the plasma membrane of the follower cell forward by utilizing a combination of actomyosin-based contractility, but membrane protrusions were generally unstable cell–cell contacts [41,42].

To quantify the level of cell polarization, we fixed cells and labeled the F-actin filaments via the Rhodamine Phalloidin (Figure 2E). Interestingly, we found that the orientation of subcellular F-actin in migrating cells depended on the distance from the leading edge of the monolayer. The self-organized actin fibers were inclined to align along a preferential direction of the front-to-rear polarity axis over time (Figure 2E,F). The self-organized actin fibers tended to align along a preferential direction of the front-to-rear polarity axis over time (Figure 2E,F). The cells close to the monolayer edge exhibited a higher degree of F-actin polarization than cells far from the edge, displaying the spatial polarization gradient. Our results suggested that the highest F-actin polarization cells located within a range of about 0–400 µm from the edge (Figure 2E,F). The polarized actin filaments assembled in a highly organized network were required for membrane protrusion formation, which initiated the movement of the follower cells. Actin assemblies can produce cytoskeletal flows that drive cell motion. By measuring cell speed, we found that the motion of the MDA-MB-231 cells was slowed down as the distance from the leading edge increased (Figure 2G,H). These results show that cancer cells are able to be polarized collectively to achieve coordinated migration, even though they have rare cell–cell junctions. The leader cells can induce the polarization of the follower cells and transmit polarity information by unstable membrane protrusion structures.

### 3.3. Collective Cell Polarization is Destructed by BFA and ML141

Collective cell polarization provides the intercellular and intracellular signals that guide dynamical processes at the cellular and tissue levels. Undoubtedly, the intercellular signal is converted into an intracellular signal that induces a great flexibility response, which is essential for the coordination of collective cancer cell polarization. To investigate the role of intracellular signal pathways in the long-range polarization of cancer cells with low intercellular interactions, we treated MDA-MB-231 cell monolayer with inhibitors.

We used Brefeldin A (BFA), which is a usable inhibitor to disrupt the Golgi apparatus polarization [43], to reduce the activity of the Golgi apparatus trafficking pathways. The BFA reversibly blocks ADP-ribosylation factor (Arf1) activity through binding to the Arf1-GDP-Sec7 complex, promoting conformational changes in Arf1, and preventing the exchange of GDP to GTP [44,45,46]. The elements Arf1 and Sec7 are essential components of the COPI coat complex of cargoes from the Golgi apparatus, which are directly transported to the ER [44,47]. The cumulative effect of BFA treatment leads to the dissociation of the Golgi apparatus and inhibition of intracellular secretory trafficking. In addition, to disrupt the activation of the Rho GTPases of the Rho family, ML141 (CID-2950007), an antagonist of the Rac1/Cdc42 signaling pathway [48], was utilized. ML141 has been demonstrated to be a potent, selective and reversible non-competitive inhibitor of Cdc42 activation in MDA-MB-231 cells [49], human ovarian carcinoma cell lines (OVCA429 and SKOV3ip) [48], and pancreatic cancer cell lines (BxPC-3, Panc-1 and HPAF II) [13]. Deactivation of Cdc42 protein activity with the inhibitor ML141 would cause a decrease in actin filaments distribution in the cortex and an impairment of cell polarization. For drug treatment, MDA-MB-231 cells were pretreated with DMSO (ctrl), BFA (5 μM), or ML141 (10 μM) for 2 h.

**Figure 3:**
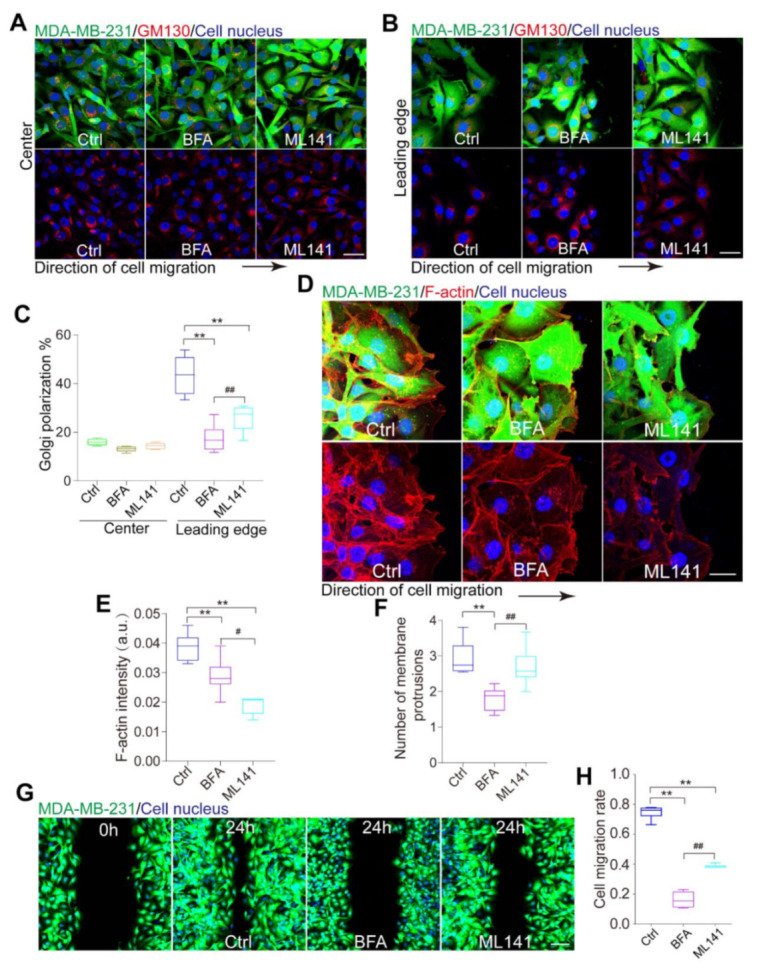
BFA and ML141 inhibit collective polarization MDA-MB-231 cells. (**A**,**B**) Drug treatment. MDA-MB-231 cells were stained for the Golgi marker GM130 (red) and cell nuclei (blue). MDA-MB-231 cells were stained for the Golgi marker GM130 (red). MDA-MB-231 cells localized at the center (**A**) and at the leading edge (**B**) of the monolayer. Scale bar, 40 µm. (**C**) The ratio of cell polarization in MDA-MB-231 cells with or without drug treatment. (**D**) Fluorescence images of F-actin (phalloidin) in migrating MDA-MB-231 cells pretreated with DMSO (ctrl), BFA (5 μM), or ML141 (10 μM) for 2 h at the leading edge. Scale bar, 40 µm. (**E**) Fluorescence intensity profile of F-actin shown in (**D**). Scale bar, 40 µm. (**F**) Calculation of the number of membrane protrusions per cell between the leader cells and follower cells. (**G**) Migration of MDA-MB-231 cell pretreated with DMSO (ctrl), BFA (5 μM), or ML141 (10 μM) for 2 h. (**H**) Quantification of relative closure of the wound shown in (**F**).Scale bar, 100 µm. Data were the mean ± SEM. #, ** and ##, *p* < 0.005, *p* < 0.001 and *p* < 0.001. *P* values were calculated using each-pair Student’s *t*-test.

We tested whether collective cell polarization could be compromised by both of the inhibitors. For Golgi apparatus orientation toward the leading edge relative to the nucleus, we found cells treated with either BFA (5 μM) or ML141 (10 μM) could lead to a decrease in the ratio of Golgi polarization at the leading edge of the monolayer, while the BFA treatment displayed more marked inhibiting effect than the ML141 treatment (Figure 3A–C). Next, we analyzed the actin fluorescence intensity at the leading edge. Our results showed that the fluorescence intensity of the ML141 treated group was significantly lower than that in both the control and the BFA treated groups (Figure 3D,E). To confirm the transmission of polarity information, the average number of membrane protrusions was calculated. We found that treatment of BFA suppressed the formation of membrane protrusions between the leader and follower cells (Figure 3D,F). During the wound healing assay, the treatment of BFA resulted in a sharp decrease in cell migration rate (Figure 3G,H). These results demonstrate that collective MDA-MB-231 cell polarization could be damaged by both BFA and ML141, whereas their inhibitory effects are different. We show that the dissociation of the Golgi apparatus by BFA received a better inhibiting effect. Collectively, we conclude that the Golgi apparatus pathways, instead of the Rac1/Cdc42 signaling pathway, are likely to play a more effective role in regulating the collective polarization of carcinoma cells.

### 3.4. Polarized Golgi Apparatus Regulates Collective Polarization of MDA-MB-231 Cells

Golgi apparatus is a crucial intracellular organelle that functions as the major protein modification and as a hub for protein transporting and delivering [32,50]. Moreover, the Golgi apparatus is also recognized as an organelle where newly synthesized proteins are post-translationally modified for intracellular trafficking [51,52], facilitating the delivery of polarity proteins toward specific regions of the cell membrane. The biosynthetic pathway by the Golgi is primordial to the secretion of extracellular matrix components, as well as the transmembrane and membrane-bound proteins, to the cell membrane [52,53]. It has long been identified that dynamic maintenance of cell polarity can be achieved through four distinct approaches: (i) activation gradients of RhoGTPases, (ii) actin cytoskeleton and focal adhesions, (iii) microtubule network and intracellular organelles, and (iv) lipid gradients and cell membrane [32]. There are many cases of interconnections between intracellular processes that highlight the complexity of cell polarization. Moreover, increasing evidence shows that cancer cells tend to employ the microtubule network and intracellular organelles as the main approach to gain collective cell polarization.

**Figure 4:**
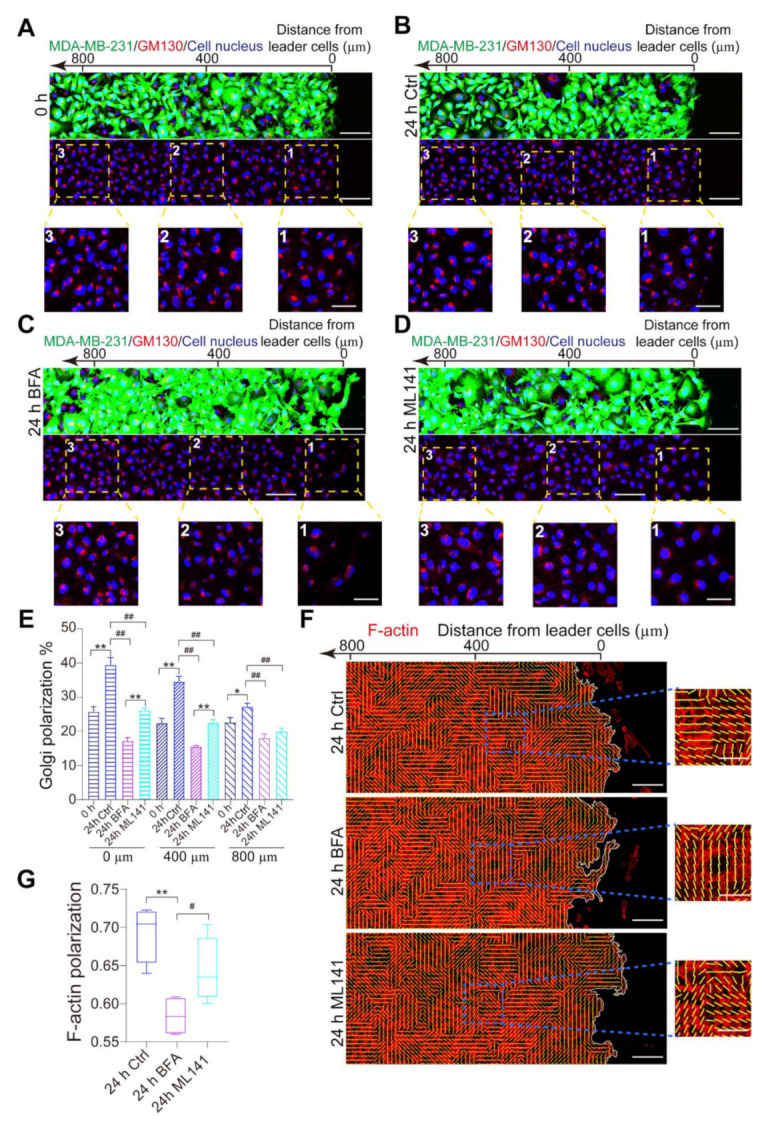
Intracellular trafficking regulates collective polarization of MDA-MB-231 cells. (**A**–**D**) Immunofluorescence images of the Golgi apparatus (GM130, red) and the nucleus (blue) in MDA-MB-231 cells at 24 h after migration. The upper images showed a wide-view field, and the lower images were magnified images of the regions corresponding to the numbered windows in the upper images. 0 h (**A**), 24 h (**B**), 24 h and BFA treatment (**C**), 24 h and ML141 treatment (**D**). Scale bar, 100 µm. The yellow dotted boxes showed a group of MDA-MB-231 cells. Scale bar, 50 µm. (**E**) The ratio of cell polarization in MDA-MB-231 cells located at a different distance from the leading edge. (**F**) F-actin cytoskeleton (red) reorganization and cell polarization in wound healing assay at 0 h and 24 h. For drug treatment, MDA-MB-231 cells were pretreated with DMSO (ctrl), BFA (5 μM), or ML141 (10 μM) for 2 h. Scale bar, 100 µm. The blue dotted boxes showed the selected area of F-actin polarized in MDA-MB-231 cells. Scale bar, 50 µm. (**G**) The ratio of polarized F-actin in MDA-MB-231 cells (0–800 µm). *, #, ** and ##, *p* < 0.005, *p* < 0.005, *p* < 0.001 and *p* < 0.001. *P* values were calculated using each-pair Student’s *t*-test.

To confirm the main pathway of MDA-MB-231 cell polarization at the population level, we utilized inhibitors to block intracellular cell polarization signal pathways. According to Figure 3E, the formation of membrane protrusions would be blocked by the treatment of BFA. The results implied that the Golgi secretory pathway takes part in the formation of membrane protrusions. For the Golgi apparatus oriented toward the leading edge, the ratio of polarized cells at the leading edge (0 µm) was higher than that at a long distance from the boundary (400 and 800 µm). The results demonstrated that the polarization signal gradually decreased with distance from the leader cells (Figure 4A–E). Pretreatment with BFA, we found the difference of polarized cells between the leading edge and the center was sharply decreased (Figure 4C,E). Although ML141 treatment could inhibit the Golgi apparatus polarization, we found a weak effect on collective cancer cell polarization (Figure 4D,E). The polarization of the Golgi apparatus in migrating cells displayed a marked spatial gradient, where cells at the leading edge of the monolayer exhibited higher polarization than cells in the monolayer center (Figure 4A–E). Compared with ML141 treatment, our results indicated that the ratio of polarized cells at the leading edge was sharply decreased by BFA (Figure 4C–E). Furthermore, we found the ratio of polarized F-actin was markedly decreased with the treatment of BFA (Figure 4F,G). Thus, unlike the Rac1/Cdc42 signaling pathway functioning in epithelial cells, the disruption of the Golgi apparatus trafficking pathways would significantly disturb the collective cancer cell polarization. Collectively, we conclude that the polarized Golgi apparatus and the secretory pathway are the predominant elements to regulate the collective polarization of MDA-MB-231 cells.

## 4. Discussion

In summary, we show that even with weak cell–cell interactions, carcinoma MDA-MB-231 cells exhibit collective polarization. By unstable membrane protrusion structures, cancer cells are able to collectively polarize to achieve concerted migration. Different from the activation Rac1/Cdc42 signaling pathway working in healthy epithelial cells, the Golgi apparatus trafficking pathways and the polarized Golgi apparatuses serve as the predominant factors in regulating collective polarization of MDA-MB-231 cells. Future studies about how collective cancer cell polarization downstream signaling pathways can be selectively blocked in tissues could lead to better therapeutic strategies for patients with invasive epithelial carcinoma.

It was reported that a strong correlation exists between malignancy and loss or disruption of cell–cell junctions in the epithelial organization [54]. In general, cancer cells that display a mesenchymal shape or spindle-like morphology are not well polarized. They contact the neighboring cells loosely and tend to be highly metastatic. Thus, loss of stable intercellular junctions and alterations in cell polarization are specific features of epithelial cancer cells. In general, most human cancer cells derived from epithelial tissues gradually lose their polarized morphology and acquire a mesenchymal phenotype [55,56]. It also indicates that the main signal pathway in collective cancer cell polarization is different from that in the healthy epithelia. During collective cancer invasion, the Golgi apparatus trafficking pathway may be a reasonable choice for the establishment of a front-to-rear polarity in cancer cells.

Golgi apparatuses are key for sorting and intracellular delivering newly synthesized proteins that are post-translationally modified, and are also necessary for intracellular cross-talking along the polarity axis of the cell. Further, cell polarization depends on directional protein transporting along Golgi-nucleated microtubules toward the front of the cell. Disrupted the structure of the Golgi apparatuses, general protein transporting to the cell surface would be interrupted and the development of membrane protrusions such as the dendritic filopodia-like precursors is prevented. It is that with the principal membrane protrusions, the polarity information can be disseminated between neighboring cells, leading to the gradient polarization in the cancer cell monolayers. It has been reported that Golgi apparatus structures activate G protein-coupled receptors by a conserved KDELR → Gαo → Rab1/Rab3 pathway, which is essential for the outgrowth of membrane protrusions [57,58].

It should be pointed out that apart from disrupting the Golgi apparatus trafficking pathways, BFA could block a wide variety of membrane transporting systems. Through efficiently preventing activation of Arf1, BFA eventually inhibits intracellular protein transport vesicles, including secretory vesicles and AP1/clathrin-coated vesicles from the *trans*-Golgi network (TGN), COPI-coated vesicles from the ER–Golgi intermediate compartment (ERGIC) [59,60,61]. However, it is hard to fully distinguish the functions of BFA between disrupting Golgi apparatus polarization or inhibiting other membrane vesicles’ elements related to cell polarization, which needs efforts to further clarify. We adopted ML141 to specifically inhibit the local activation of Rho family GTPases (Cdc42), as commonly used in previous studies [48,62]. Nevertheless, the transport of Cdc42 via the Golgi apparatus could be impaired slightly by BFA. As small molecules, the activation of Cdc42 controls the spatiotemporal cell polarization mainly associated with the actin cytoskeleton [63,64]. Our data showed that in controlling collective cancer polarization, the Golgi apparatus pathways predominate over the Rac1/Cdc42 signaling pathways.

In the present study, we consider the polarization of two-dimensional cancer monolayers *in vitro*. However, most solid tumors *in vivo* have three-dimensional structures. Such three-dimensional structures lead to different cell–cell contacts and interactions, which would affect intercellular communications and, thus, collective cell polarization. Moreover, *in vivo* cancer invasion commonly undergoes interactions between cancer cells and host tissues, as well as cell–ECM interactions, which are absent in our monolayer assay. The polarization and motility of cancer cells may be modulated by the neighboring healthy cells and microenvironmental conditions. These issues merit future experimental studies.
